# Water extract from processed *Polygonum multiflorum* modulate gut microbiota and glucose metabolism on insulin resistant rats

**DOI:** 10.1186/s12906-020-02897-5

**Published:** 2020-04-05

**Authors:** Wen Gu, Min Yang, Qian Bi, Lin-Xi Zeng, Xi Wang, Jin-Cai Dong, Feng-Jiao Li, Xing-Xin Yang, Jing-Ping Li, Jie Yu

**Affiliations:** College of Pharmaceutical Science, Yunnan University of Traditional Chinese Medicine, 1076 Yuhua Road, Chenggong District, Kunming, Yunnan Province People’s Republic of China

**Keywords:** Glucose metabolism, Gut microbiota, Insulin resistance, Processed *Polygonum multiflorum*

## Abstract

**Background:**

The incidence of insulin resistance (IR) has rapidly increased worldwide over the last 20 years, no perfect solution has yet been identified. Finding new therapeutic drugs will help improve this situation. As a traditional Chinese medicine, PPM (processed *Polygonum multiflorum*) has widely been used in the clinic. Recently, other clinical functions of PPM have been widely analyzed.

**Results:**

Administration of the water extract from PPM decreased the level of FBG, TC, and TG, and increased the level of FGC, thereby reducing the IR index and improving IR. Furthermore, Western blot analysis revealed that PPM significantly increased GPR43 and AMPK expression when compared with the MOD group, and GPR43, AMPK were known as glucose metabolism-related proteins. In addition, treatment with PPM can restore the balance of gut microbiota by adjusting the relative abundance of bacteria both at the phylum and genus level, and these changes have been reported to be related to IR.

**Methods:**

Sprague Dawley (SD) rats were fed a high-fat diet and were gavaged daily with either normal saline solution or PPM for 12 weeks. Major biochemical indexes, such as fasting blood glucose (FBG), fasting glucagon (FGC), total cholesterol (TC), and triglyceride (TG) were measured. Then the protein expression of adenosine 5′-monophosphate -activated protein kinase (AMPK) and G protein-coupled receptor 43 (GPR43) was evaluated by using Western blot analysis. Moreover, the composition of gut microbiota was assessed by analyzing 16S rRNA sequences.

**Conclusions:**

Our findings showed that PPM reversed the increasing of FBG and the decreasing of IRI, PPM accelerated the expression of glucose metabolism-related proteins and regulated the intestinal microecological balance. Therefore, we hold the opinion that PPM may be an effective option for treating IR.

## Background

IR, as the basis of the onset of type 2 diabetes, is defined as the reduced ability of insulin-sensitive tissues to respond to insulin, and plays a key role in the development of metabolic diseases [1, 2]. IR has attracted increased attention due to its annual increase in prevalence [3–5]. Direct causes of IR have not yet been identified, however, it is known that exposure to a prolonged high-fat diet and an irregular diet result in IR in both humans and animal models [6]. Recent studies have suggested that dyslipidemia and pathoglycemia closely correlate with IR [7–10].

PPM (originated from the processed root of *Polygonum multiflorum* Thunb.), a well-known classical traditional Chinese medicine, has high medicinal value. At present, PPM is widely used in the treatment of dyslipidemia and the similar diseases [11–13]. In our previous studies, the lipid-lowering activity of PPM was investigated in animal models of non-alcoholic fatty liver disease (NAFLD) [14].

On the other hand, tetrahydroxy stilbene glucoside (TSG), which is an active component of *P. multiflorum,* was found to be effective in HFD-induced CF-1 diabetic mice [15]. Subsequent research indicated that TSG could inhibit of the tissue renin-angiotensin system (RAS), which plays a critical role in development of diabetic osteoporosis [16].

Our previous research [17] also suggested TSG may improved intestinal micro-ecological disorders, which may be involved in the initial of IR [18, 19]. These clues indicated that PPM may not only regulate the lipid metabolism disorder, but also regulate the body’s glucose metabolism and IR. However, no relevant research has been found, efficacy and the mechanism are still unclear.

In the present study, we systematically evaluated the effect of PPM in high-fat diet-induced IR rats, and examined possible mechanisms of PPM. We found that PPM improved IR. We also elucidated the changes in gut microbiota and critical glucose metabolism-related proteins in PPM treatment. In this study, we provided a novel treatment of IR, which may lead to a further improvement of IR therapy.

## Methods

### Chemicals and materials

Ethanol, petroleum ether, chloroform, and n-butanol were purchased from Tianjin (Sailing Chemical Reagent Technology Co., Ltd., China). Metformin hydrochloride (MET), used as positive control [20], was purchased from Shenzhen (Haiwang Pharmaceutical Co., Ltd., China). Water was purified using a Milli-Q system (Millipore, Bedford, MA, USA). Glucose test strips (ACCU-CHEK Performa test strips) were purchased from Roche Diagnostics GmbH Co., Ltd., China. Edible lard was purchased from Sichuan Green Island Grease Co., Ltd. (Sichuan, China). Sucrose was purchased from Tianjin Sailing Chemical Reagent Technology Co., Ltd. (Tianjin, China). Cholesterol was purchased from Beijing Boao Extension Technology Co., Ltd. (Beijing, China).

### Processing and extraction of PPM

*Polygonum multiflorum* Thunb. was collected from Luquan county, Yunnan Province, and the raw materials were authenticated by Professor Ronghua Zhao of the Yunnan University of Chinese Medicine (Specimen number: BQ 20160701). Voucher specimens were deposited in the Herbarium of Pharmacognosy, Yunnan University of Chinese Medicine. PPM was prepared in our laboratory according to the Chinese Pharmacopoeia (Chinese Pharmacopoeia, 2015). At first, the roots of *Polygonum multiflorum* Thunb. were thickly sliced (5-9 mm). Then, 1 kg sliced root was mixed and steamed with black bean juice (0.25 kg) for 2.5 h. Then, they were dried at 60 °C in the oven for further use.

PPM (1000 g) was decocted with 10 L ultrapure water for 1 h at 100 °C. Then, residues were decocted again with 8 L, 6 L ultrapure water for 40 min at 100 °C, respectively. Filtrates were combined and extraction was condensed and lyophilized to yield 41.3% of product.

According to Chinese Pharmacopoeia, the usual clinic doses of PPM for humans was 9 g/kg per day. We converted it to the middle dosage of rats. Based on the dose conversion relationship between humans and rats according to the body surface area (Rat dose = human dose g*0.018/0.02 kg), the middle dose of raw PPM materials for rats was set for 0.81 g·kg^− 1^. Correspondingly, high and low doses of raw PPM materials for rats were 0.405 and 1.62 g·kg^− 1^, respectively.

### Animals and experimental design

Five–Six weeks old Male Sprague Dawley (SD) rats, weighing of 180 ± 20 g, were purchased from Sichuan (Dashuo Laboratory Animal Technology Co., Ltd., Chengdu, China). Rats were housed in a controlled environment (temperature of 22–25 °C; relative humidity of 50% ± 5%) and kept on a light/dark cycle of 12/12 h. Rats were fed a standard commercial rat diet (Suzhou Shuangshi Experimental Animal Feed Technology Co., Ltd.) or high-fat diet (containing 79% base feed, 10% lard, 10% sucrose, and 1% cholesterol).

After 3 days of acclimatization, rats were randomly divided into 5 groups of 10 rats each: control group (CON), model group (MOD), positive control group (MET), water extraction group of PPM (low, and high dose groups: PPM-L, PPM-H). CON group rats were fed with standard commercial rat diet, and other rats were fed with high-fat diet until the end of the experiment. Rats in the CON group and MOD group were given normal saline daily, while rats in other groups received MET or PPM daily, respectively.

**Fig. 1:**
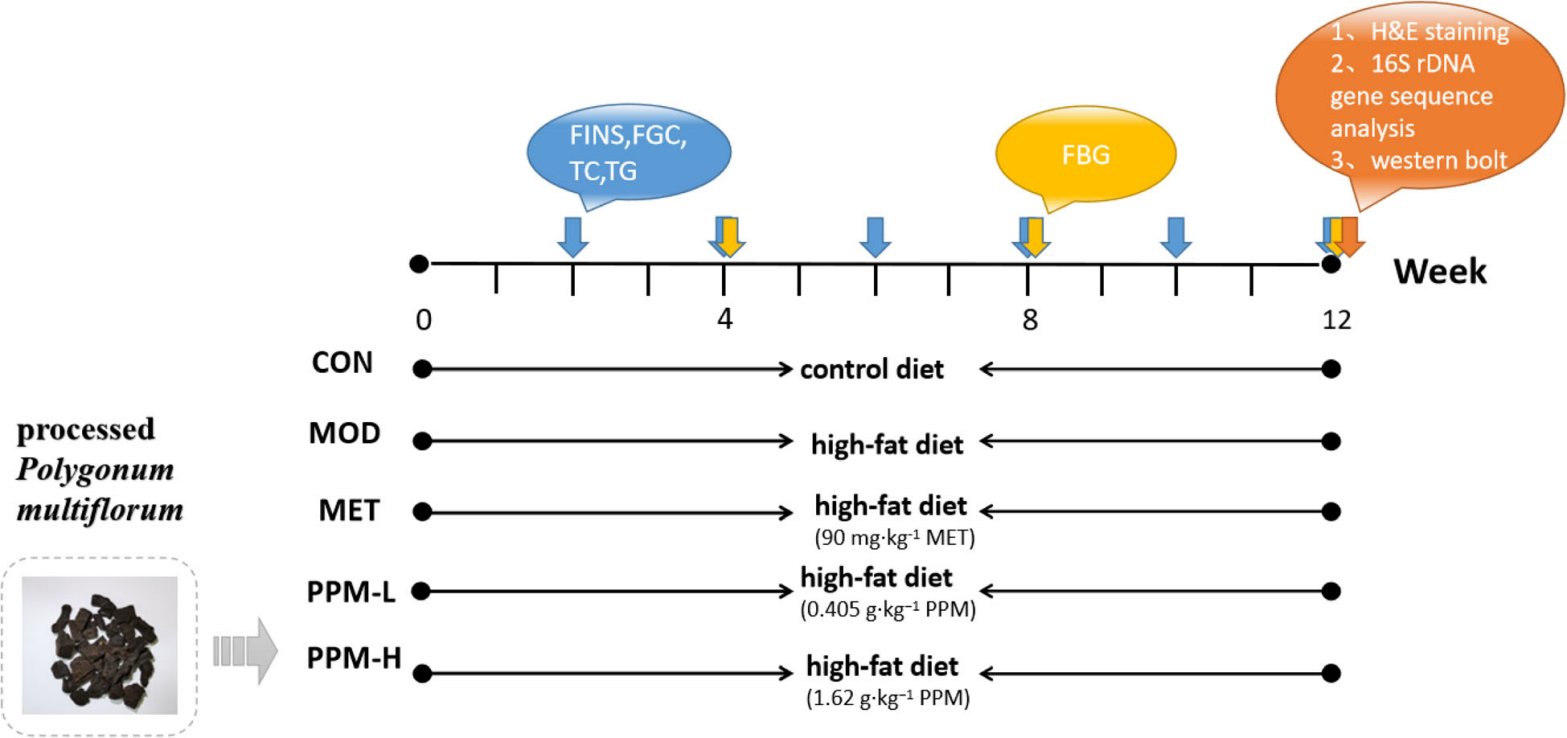
Experimental design. After 3 days of acclimatization, rats were randomly divided into five groups: control group (CON), model group (MOD), positive control group (MET), water extraction group of PPM (low high dose groups) (PPM-L) and water extraction group of PPM (high dose groups) (PPM-H). Except for the CON group, all rats received a high-fat diet for 12 consecutive weeks. Rats were fasted for 2 h each day prior to administration of therapeutic agents. In the CON and MOD groups, rats were gavaged with normal saline, whereas in the MET group, rats were gavaged with 90 mg·kg^− 1^ Metformin hydrochloride, in the PPM-L group, rats were gavaged with 0.405 g·kg^− 1^ PPM, and in the PPM-H group, rats were gavaged with1.62 g·kg^− 1^ PPM

Moreover, the MET group received 90 mg·kg^− 1^ MET daily. PPM-L (0.405 g·kg^− 1^) and PPM-H (1.62 g·kg^− 1^) groups received PPM treatments for 12 consecutive weeks until the end of the study. All rats were fasted for 2 h every day before administration of therapeutic agents. Rats in each group were weighed once a week for 12 consecutive weeks (Fig. 1).

At the end of the experimental period, rats were anesthetized with sodium pentobarbital (40 mg/kg) administered intraperitoneally and euthanized by cervical dislocation. The animal procedures were approved by the Animal Experiment Ethics Committee of Yunnan University of Traditional Chinese Medicine (Approval Number: R-0620160030). Rats were also handled according to the guidelines set by the National Institutes of Health (NIH). Then, rats were quickly dissected to remove the liver, pancreas and adipose tissue.

### Analysis of pharmacological effects of PPM

Rat blood samples (1.5–2 mL) were collected from the ocular vein every 2 weeks. Blood was centrifuged (10,000 rpm, 15 min) and supernatant was collected for future assays. Contents of fasting insulin (FINS) and FGC were determined by an ELISA assay kit purchased from Hubei (Huamei Co., Ltd., Wuhan, China) and contents of TC and TG were determined using a kit that was purchased from Beijing (Zhongsheng Beikong Co., Ltd., China) following the manufacturer’s guidelines. The blood glucose meter (ACCU-Check blood glucose meter, Roche Diagnostics, Shanghai, China) is used by gently prick the tail of the rat, and the FBG levels of rats and blood will be quickly measured in the 4th, 8th, and 12th week. The IR index (IRI) was calculated using the following formula: IRI = (FBG* fasting insulin) / 22.5. Oral glucose tolerance test was performed 1 day before the end of the experiment. The rats were given 50% glucose solution and obtained blood samples via the tail vein, the blood glucose values were measured sequentially with blood glucose meter at 0 h, 0.5 h, 1 h, 1.5 h and 2 h after glucose dosing. Draw the oral glucose tolerance test (OGTT) curve and calculate the area under the curve.

The liver of the same part of each rat was collected for fixation, and the remaining part was used to protein assay. The liver was fixed in 10% formaldehyde solution for 24 h. Then, the tissues were embedded in paraffin. Serial 5 μm sections were cut and stained with hematoxylin and eosin. The staining results were visualized by microscopy.

### Analysis of glucose metabolism related proteins

The protein expression of GPR43 and p-AMPK was determined by Western blot analysis. In brief, liver and muscle tissues (100 mg) from each rat were homogenized in 1 mL PIRA lysis buffer, then centrifuged at 12,000×g for 10 min at 4 °C. Samples were kept on ice for 30 min the supernatant was collected. Before gel electrophoresis, total protein concentration was determined using the bicinchoninic acid (BCA) method. Total protein (40 μg) was separated by sodium dodecyl sulfate-polyacrylamide gel electrophoresis (SDS-PAGE) and transferred to a polyvinylidene fluoride (PVDF) membrane. Albumin bovine V (BSA, 5%) was prepared in TBS containing Tween-20 (TBST), and samples were incubated in 5% BSA for 2 h at 24 °C to block non-specific proteins. Then, membranes were incubated overnight at 4 °C with primary antibodies (2,935,448, 1:1000 dilution; EMD Millipore Corp., United States) and GAPDH (10494–1-AP, 1:10000 dilution; Proteintech Group, United States) used as an internal control. Then, membranes were washed 3 times with TBST and incubated with the secondary antibodies (SA00001–2, 1:10.000 dilution; Proteintech Group, United States) for 2 h after being washed 3 times in TBST. Proteins were visualized using an enhanced chemiluminescence (ECL) detection system (Proteintech Group, United States) and quantified with Quantity One Analysis Software (Bio-Rad Laboratories, Inc., United States).

### Analysis of gut microbiota composition

Total genomic DNA was extracted from fecal samples of 12th weeks laboratory rats using the Stool DNA Kit (Omega Corp., United States). The DNA was stored at − 20 °C for future pyrosequencing analysis of V4 regions of 16S rDNA.

PCR amplification was conducted using a 338F_806R primer set, and the V4 region of 16S rDNA gene was amplified. PCR products were quantified by Qubit@ 2.0 Fluorometer (Thermo Fisher Scientific Corp., United States) and Agilent Bioanalyzer 2100 system. Sequencing libraries were prepared by using TruSeq DNA PCR-Free Sample Preparation Kit (Illumina Corp., United States) according to the manufacturer’s guidelines and index codes were added. At last, the library was sequenced in the IlluminaHiSeq 2500 platform and 250 bp paired-end reads were generated.

### Statistical analysis

Data are expressed as the mean ± S.E.M. All statistical analyses were performed using an SPSS 16.0 software package. To identify significant differences between groups, one-way ANOVA was performed (Assuming that the variance is equal, the LSD:1 method is used for comparison. Assume that the variance is not uniform when using the Tamhanes T2 test, *p* < 0.05, *p* < 0.01, and *p* < 0.001). For the 16S rDNA gene sequence analysis findings, correlation data analyses were performed using partial least square discrimination analysis (PLS-DA), spearman correlation matrix, and hierarchical clustering tree.

## Results

### Pharmacological effects of PPM

An animal model of IR was established by feeding rats a high-fat diet. At the end of the experiment, no significant difference in body weight was observed between groups, and the blood collection procedures have no significant effect on body weight (Figure S1).

**Fig. 2:**
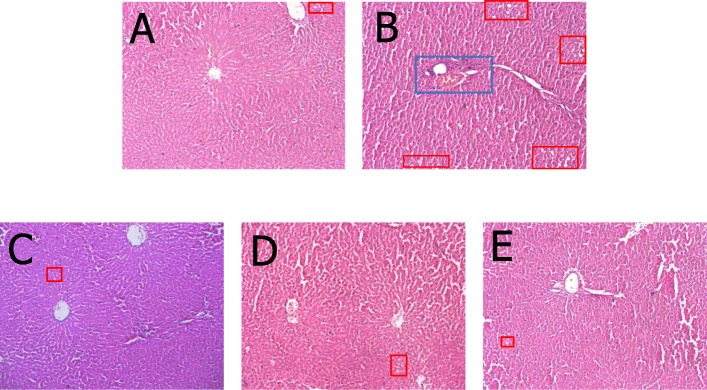
Effect of PPM in liver tissue of insulin-resistant rats.the MOD group had obvious fatty degeneration in the liver cells, while the MET, PPM group was less severe. (**a**) normal control group, (**b**) model control group, (**c**) positive control group, and (**d**、**e**) group treated with 0.405 and 1.62 g·kg^− 1^ PPM. The red box represents the lipid vacuole, and the blue box represents the portal inflammation

Liver sections from the CON group displayed a normal cell structure and lobular architecture. On the contrary, liver specimens from the MOD group showed irregular hepatocyte arrangement, edema, portal inflammation and increasing vacuolated lipid droplets. Treatment of MET, PPM relieved the steatosis of liver cells, especially in PPM-H group (Fig. 2).

**Fig. 3:**
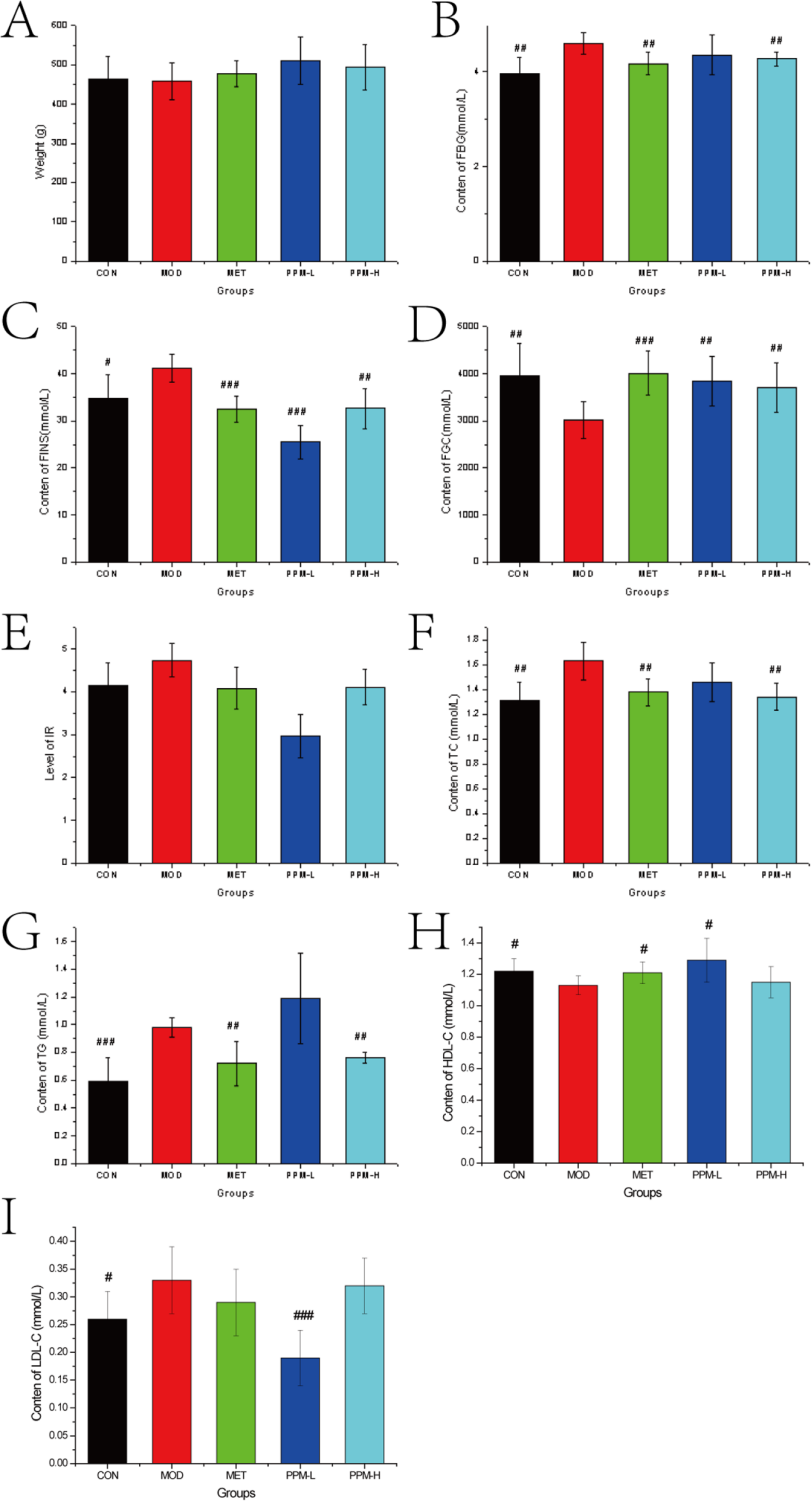
Comparison of insulin resistance index (IRI) biochemical indicators in different groups after 12 weeks of treatment with PPM. At the end of the study, significant differences were compared between groups. ^*^P < 0.05, ^**^P < 0.01, and ^***^P < 0.001 compared to the normal control group, ^#^*P* < 0.05, ^##^*P* < 0.01, and ^###^*P* < 0.001 compared to the model control group. FBG: fasting blood glucose, FINS: fasting insulin, FGC: fasting glucagon, TC: total cholesterol, TG: triglyceride, HDL-C: high-density lipoprotein cholesterol, LDL-C: low density lipoprotein cholesterol

After 12 weeks of feeding the high-fat diet, FINS, FBG, TG, and TC in the MOD group were significantly increased when compared to those in CON group, whereas FGC levels were decreased. In comparison with the MOD group, the FBG level in PPM groups was significantly decreased and tended toward normal levels. Moreover, PPM treatment also reduced the level of TC and TG, among them, the water extraction group of PPM (high dose groups: PPM-H) had a better effect on down-regulation. Together, these results indicated that PPM prevented the increase of FBG, TC, and TG in high-fat diet fed rats (Fig. 3).

As presented in Fig. 3, we found that the content of HDL-C in serum of the MOD group was significantly lower compared to that of the CON group, whereas low density lipoprotein cholesterol (LDL-C) was significantly higher when compared to that of the CON group. Thus, PPM may reduce the accumulation of TG in the liver by decreasing the content of LDL-C and by increasing the content of HDL-C.

### Effect of PPM on the expression of glucose metabolism-related proteins

**Fig. 4:**
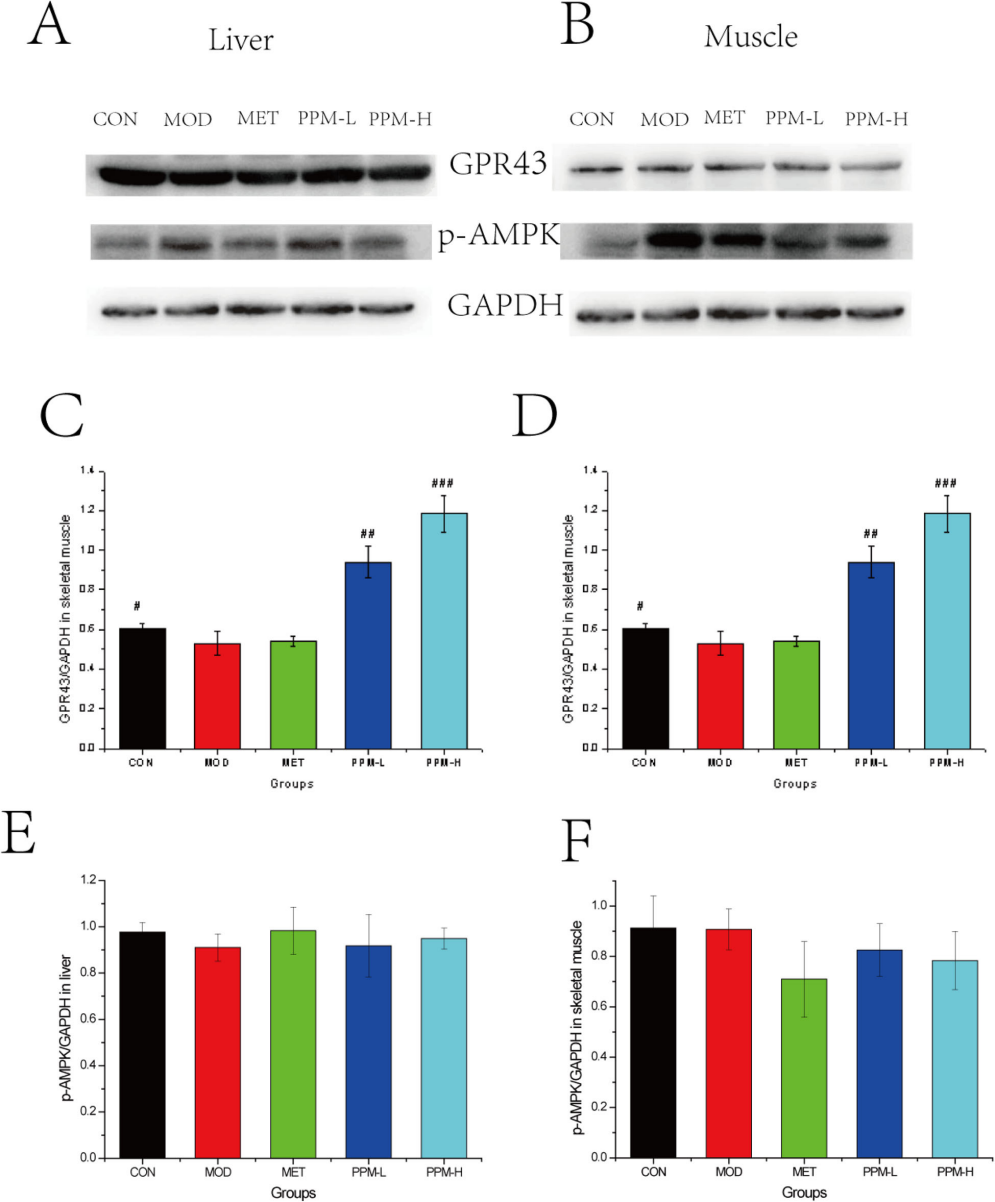
Immunoblot analysis for p-AMPK, GPR43 in liver (**a**) and muscle (**b**) after treatment with MET or PPM for 12 weeks. GAPDH was used as an internal control. In the histogram, values are presented as the mean ± S.D. (*n* = 10 rats per group). After treatment with MET and PPM, p-AMPK expression in liver and muscle increased. Increased GPR43 expression in liver and decreased GPR43 expression in muscle when compared to controls. ^*^P < 0.05, ^**^P < 0.01, and ^***^P < 0.001 compared to the normal control group, ^#^*P* < 0.05, ^##^*P* < 0.01, and ^###^*P* < 0.001 compared to the model control group

To evaluate whether PPM can affect the protein expression of GPR43 and p-AMPK, Western blot analyses were performed to explore the mechanism of PPM in rats. As shown in Fig. 4, the expression of GPR43 was significantly decreased in the MOD group when compared with the CON group, whereas in the PPM groups GPR43 protein was increased when compared with the MOD group. Treatment with PPM-H increased the GPR43 level in liver by 19.1% and in skeletal muscle by 82.8%. Moreover, the expression of p-AMPK was significantly different between CON and MOD groups. PPM-L increased p-AMPK protein expression in liver by 4.3% relative to the MOD group.

### Effect of PPM on gut microbiota composition

**Fig. 5:**
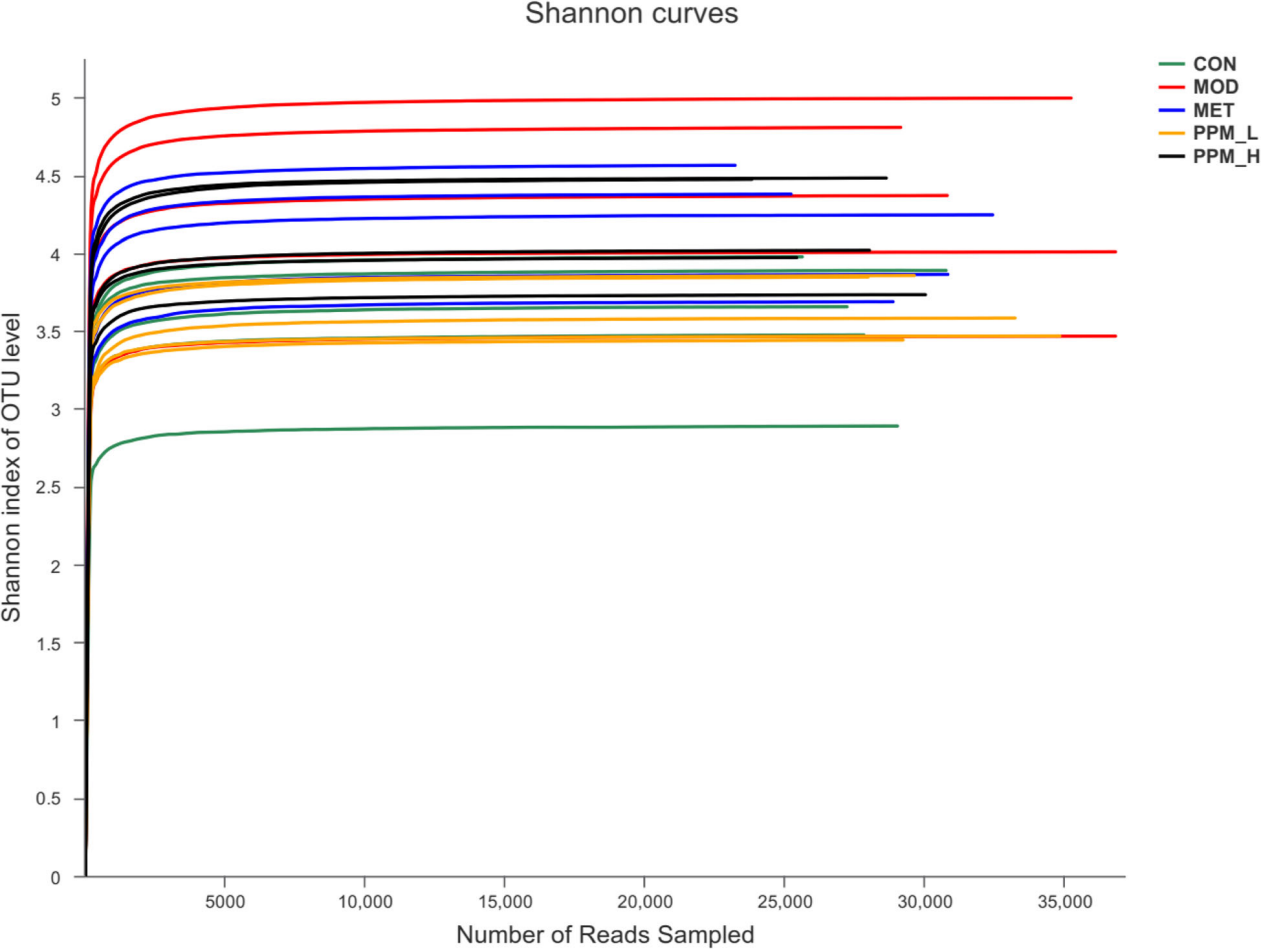
The Shannon-Wiener curve reflects the microbial diversity of each sample at different sequencing numbers. A flat curve indicates that the amount of sequencing data is large enough to reflect the vast majority of microbial information in the sample

**Fig. 6:**
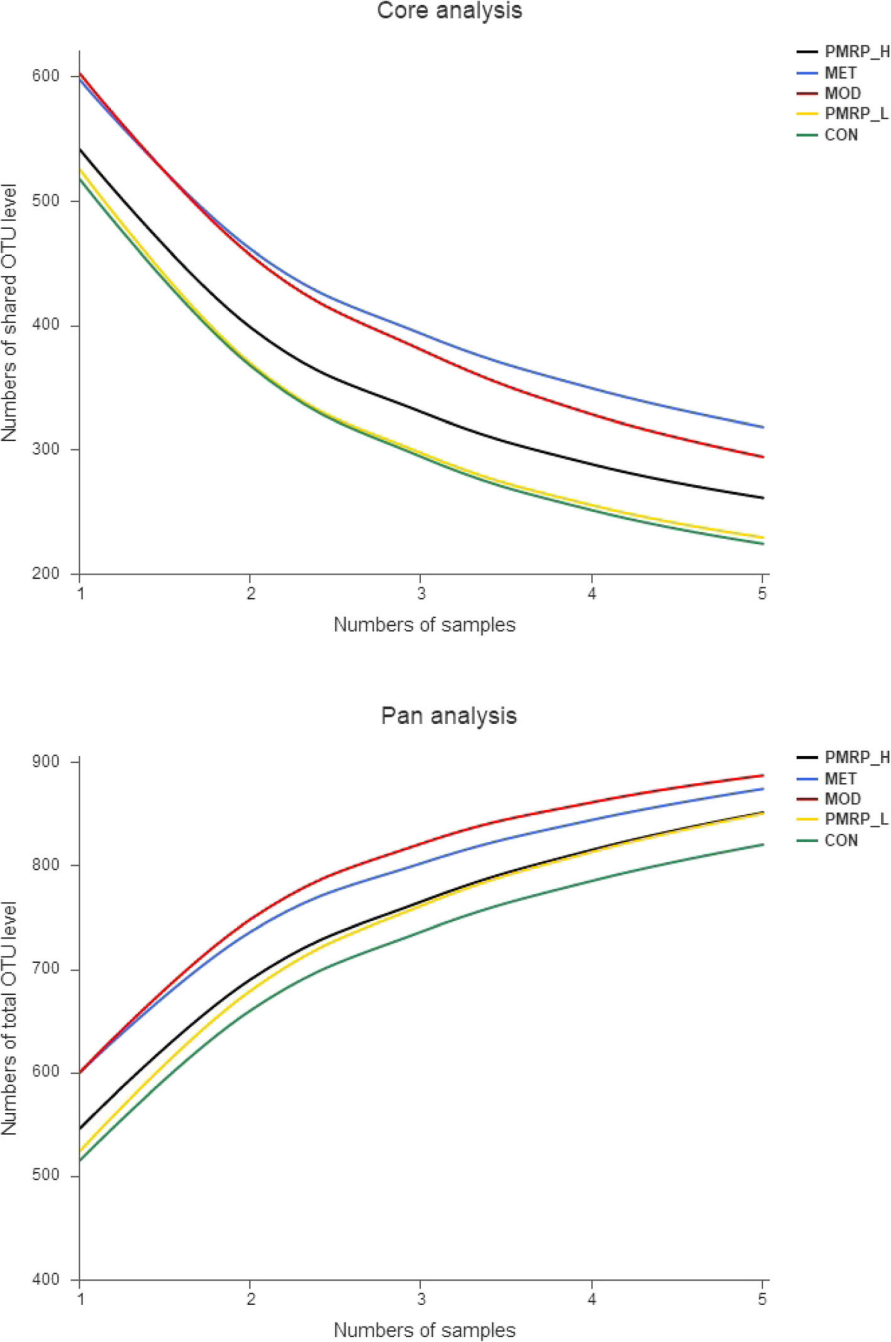
Pan-network indicates the Optical Transform Unit (OTU) of the union and increases with an increasing sample size. A core-network indicates the intersection OTU, which decreases when the sample size increases

To illustrate the feasibility and accuracy of our gut microbiological data, two methods of data analysis were used: Shannon-Wiener curves, which measure the evenness and richness of the data (Fig. 5), and pan-network and core-network representing union and intersection between a sizeable fraction of individual networks, respectively (Fig. 6). The results showed that the sequencing data were sufficient to reflect the majority of microbial information in the sample.

**Fig. 7:**
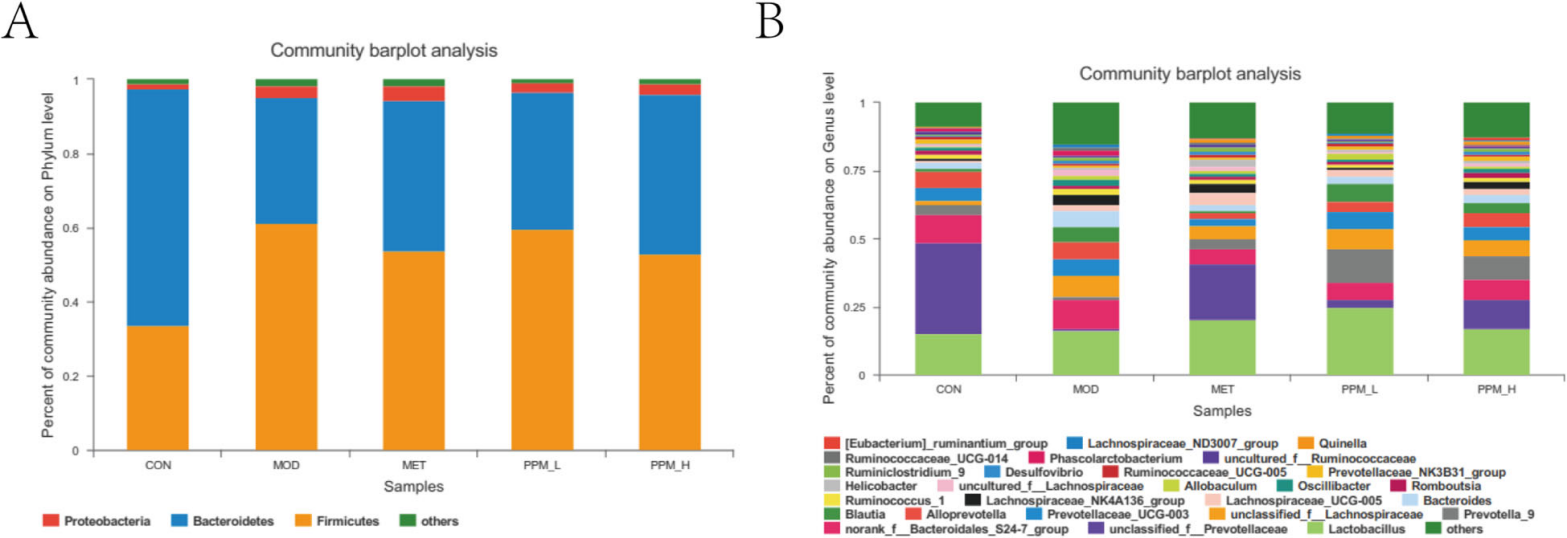
Comparison of gut microbiota in different groups. **a** Relative abundance of main phylum > 0.1% in the different groups. **b** Relative abundance of main genus in the different groups

At the phyla level, Taxonomy-based analysis indicated that Firmicutes and Bacteroidetes were the predominant phylum, followed by Proteobacteria, and an unclassified group denoted as “other”. At the taxonomic level of phylum, the ratio of Firmicutes-to-Bacteroidetes in the MOD group was significantly increased by 246.2% relative to the CON group. Moreover, the relative abundance of Baceroidetes in PPM-H treatment groups was significantly increased by 25.9%, whereas the relative abundance of Firmicutes was significantly decreased by 12.7% when compared with the MOD group (Fig. 7a).

**Fig. 8:**
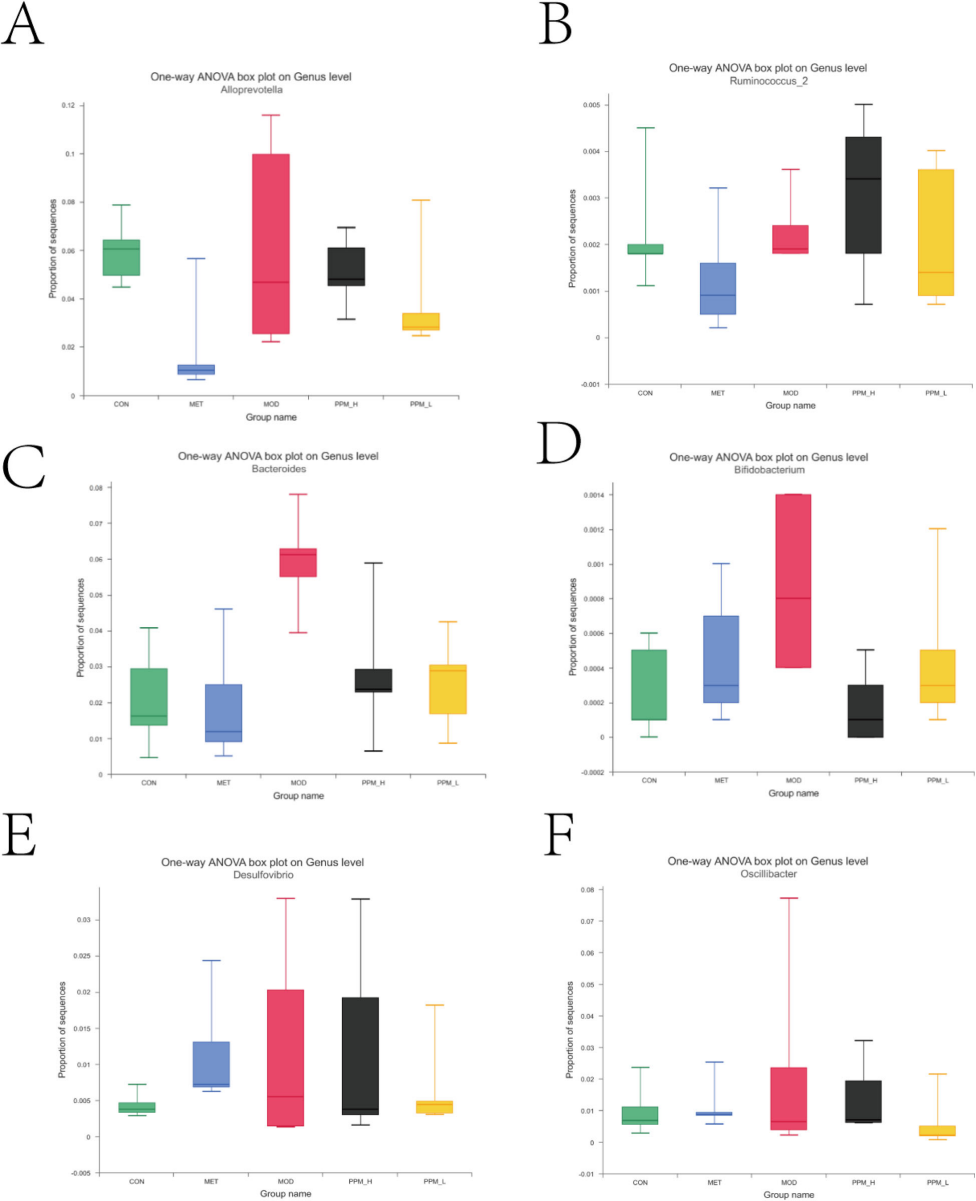
Boxplots of the abundance of genus in different groups. Treatment with PPM changed the relative abundance of gut microbiota. A-F represents the selected genus, including: **a** Alloprevotella spp., **b** Ruminococcus_2 spp., **c** Bacteroides spp., **d** Bifidobacterium spp., **e** Desulfovibrio spp., **f** Oscillibacter spp., the mid line in the boxplot represents the median

When comparing CON and MOD groups at the genus level, there was a greater increase in the relative abundance of Desulfovibrio spp., Bifidobacterium spp. and a decrease in the relative abundance of Alloprevotella spp., Ruminococcus_2 spp., Bacteroides spp. In addition, treatment with PPM increased the relative abundance of Alloprevotella spp. and decreased the relative abundance of Desulfovibrio spp. In particular, the level of two crucial and beneficial types of symbiotic bacteria, Bacteroides spp. [21] and Bifidobacterium spp. [22] was increased, while two types of pathogenic bacteria, Desulfovibrio spp. [23, 24] and Oscillibacter spp. [25] were decreased in the PPM groups (Figs. 7b and 8).

**Fig. 9:**
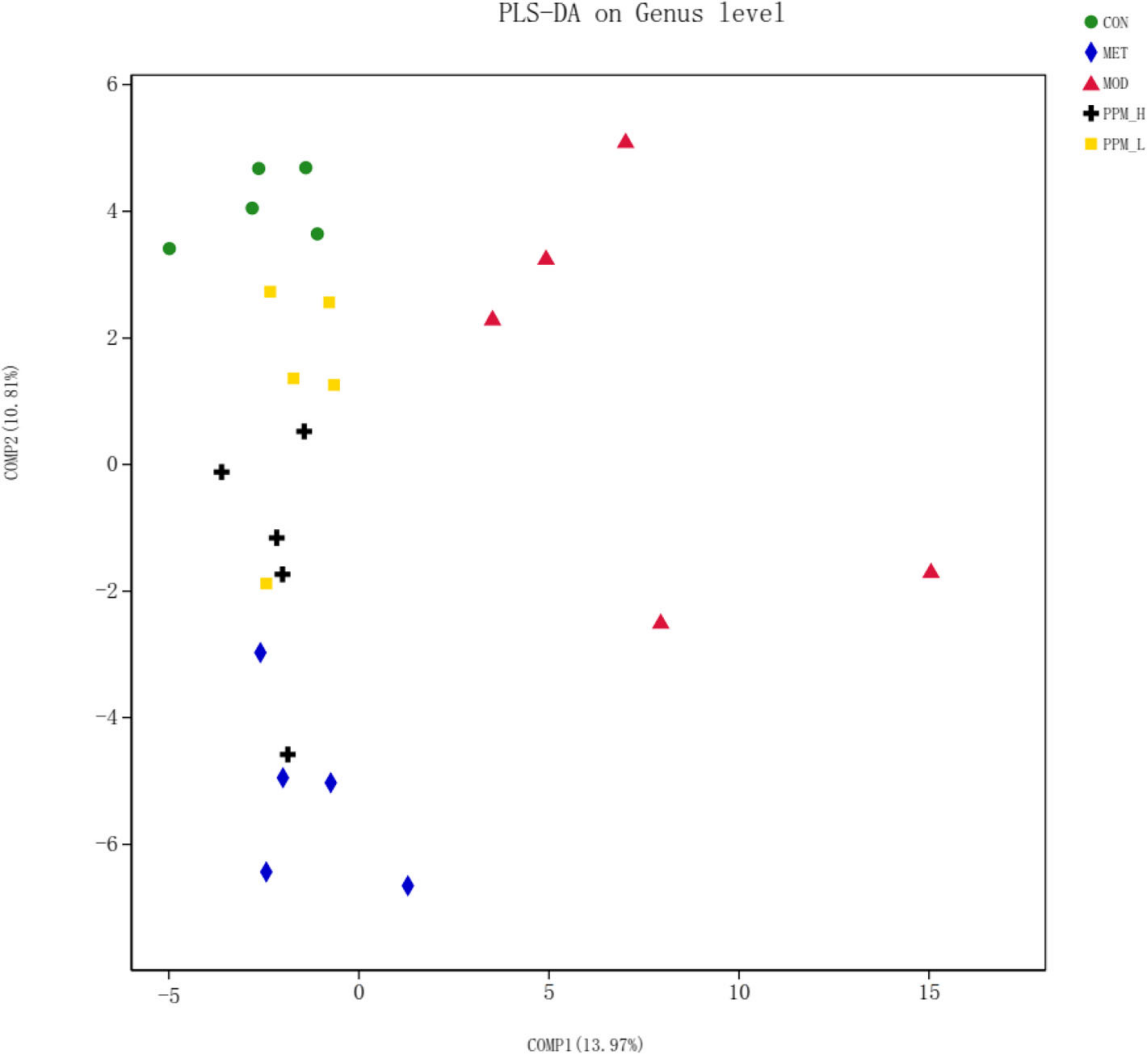
Comparison of gut microbiota in different groups. Partial least squares discriminant analysis (PLS-DA) on unweighted Unifrac distances in different groups. Each colored symbol presents the composition of gut microbiota of a single rat

To test whether the overall bacterial composition was different between experimental groups, we used partial least squares discriminant analysis (PLS-DA) on unweighted Unifrac distances. As presented in Fig. 9, significant differences were observed between the CON and MOD group, and the medicated group approached the CON group.

**Fig. 10:**
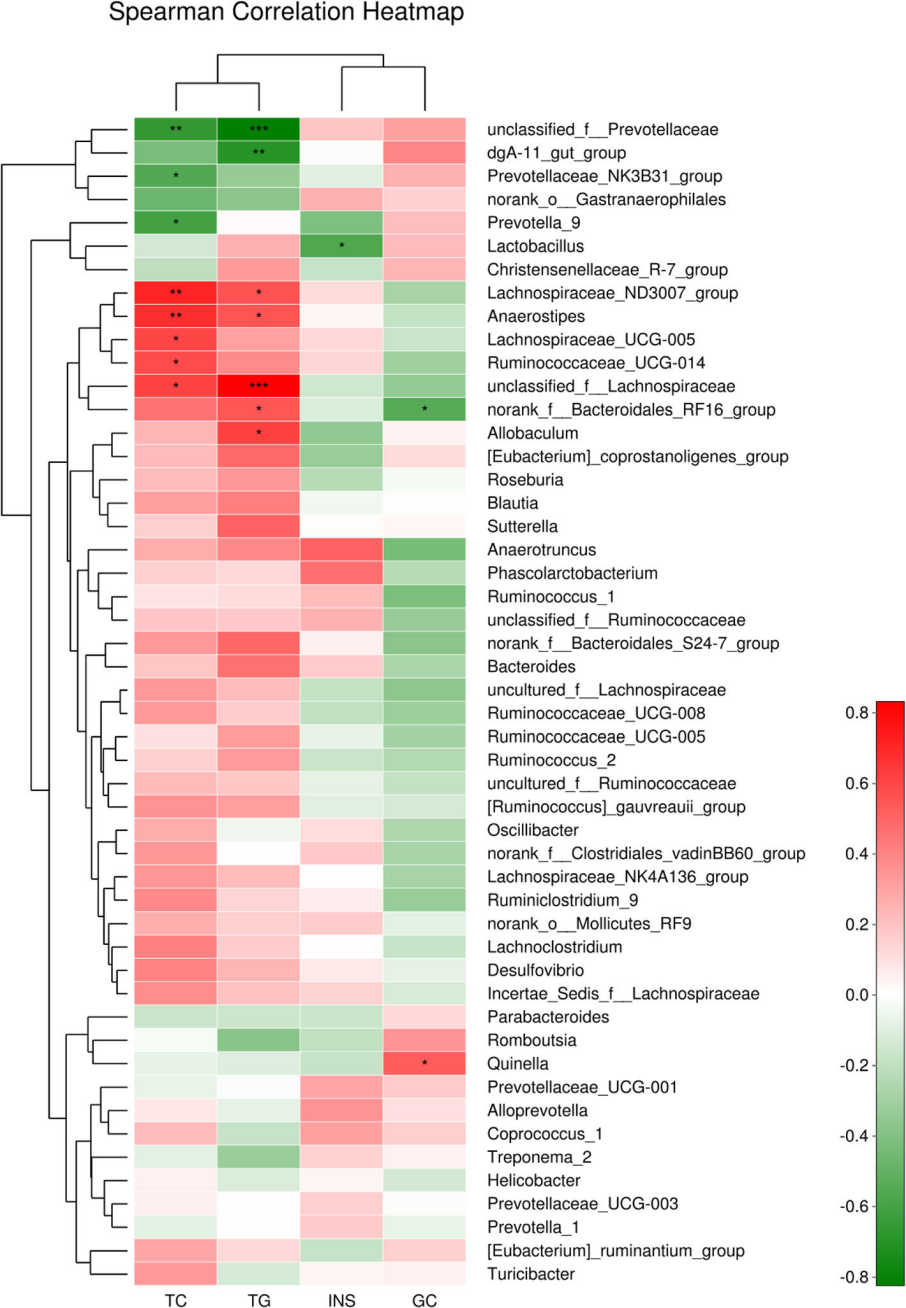
Heatmap of the microbial composition at the genus level. The heatmap indicates the relative abundance of each genus in different samples. A negative correlation is indicated in red, a positive correlation is indicated in green. The darker the color, the more significant the correlation

Figure 10 presents the heatmap that is based on the spearman correlation matrix. After data correction, the top 50 genera with different abundances were identified according to the color variations. Red represented the genus in high abundance, whereas green represented low abundance. Spearman’s correlation analysis showed that 50 key operational taxonomic units (OTUs) negatively or positively associated with IR. Lachnospiraceae, Ruminococcaceae, and Anaerostipes positively correlated with TC, whereas Prevotellaceae negatively correlated with TC. Lachnospiraceae, Anaerostipes, Bacteroidales, and Allobaculum positively correlated with TG, whereas Prevotellaceae negatively correlated with TC. Bacteroidales negatively correlated with FGC, whereas Quinella positively correlated with FGC. Lactobacillus showed a negative correlation with INS.

Overall, these findings indicated that treatment with PPM can restore the balance of gut microbiota by adjusting integrate constitution both at the phylum and genus level.

## Discussion

IR is a common metabolic syndrome disease that is associated with major public health problems worldwide [26]. Furthermore, in our previous studies, we demonstrated that PPM had a good lipid-lowering effects in vitro and in vivo [17, 27]. Therefore, we expected to unravel the effects and underlying mechanism of action of PPM in the treatment of IR.

Several previous studies demonstrated that PPM had beneficial effects in obesity and hyperglycemia [28, 29]. Here we provided evidence for the beneficial effects of PPM on an IR rat model. PPM not only reversed diet-induced hyperglycemia, but also ameliorated hepatopathy. Moreover, PPM reduced the expression of LDL-C and increased HDL-C levels, and reduced the accumulation of TG in hepatocytes, thereby indicating it was effective in alleviating IR.

GPR43 is a receptor that senses short-chain fatty acids, and is expressed in various immune and non-immune cells [30]. Studies have shown that GPR43 is related to fat accumulation [31]. Moreover, adenosine 5′-monophosphate (AMP)-activated protein kinase (AMPK) plays a major role in bioenergy metabolism and is an upstream regulator of fatty acid oxidation, hydrolysis, and lipid metabolism [32]. Activation of AMPK is thought to be a therapeutic target to recover metabolic balance caused by diabetes mellitus [33]. In the present study, Western blot analysis revealed that PPM significantly increased GPR43 and AMPK expression when compared with the MOD group. These results indicated that PPM served as an effective anti-diabetes agent by targeting restoration of GPR43, and AMPK expression.

The disruption of intestinal microecology balance is now recognized as a significant contributor towards the development of obesity and IR [34, 35]. Based on the analysis of microbial 16S rDNA gene detection, we attempted to explore the correlation of gut microbiota and PPM. Our findings demonstrated that rats in the MOD group showed increased Firmicutes and decreased Bacteroidetes when compared to the rats in the CON group. After PPM treatment, the reduction in Firmicutes abundance and the increase in Bacteroidetes abundance led to a decrease in the Firmicutes: Bacteroidetes ratio. Interestingly, several studies have shown that the Firmicutes: Bacteroides ratio was important in metabolism and related to weight [36–38]. Huang et al. found that this ratio was related to type 1 diabetes mellitus [39]. Furthermore, our study indicated that the relative abundance of two crucial symbiotic bacteria, Bacteroides spp. and Bifidobacterium spp. was different between rats in the CON and MOD group. These results were consistent with the data presented by Li et al., who found that changes in Bacteroides spp. and Bifidobacterium spp. were related to type 2 diabetes [40]. Taken together, our results showed that the abundance of gut microbiota changed after PPM supplementation, indicating that the role of PPM in alleviating IR may be potentiated by regulating the intestinal microecology.

## Conclusions

The findings of the present study indicated that PPM had therapeutic effects on diet-induced IR in rats. The protective effects of PPM in IR rats may be associated with its ability to regulate the intestinal microecological balance, thereby accelerating the expression of glucose metabolism-related proteins. These results provided evidence that PPM is a promising candidate for the treatment of IR. Further studies will be required to elucidate the underlying mechanisms of the therapeutic effects of PPM and may facilitate the identification of treatment options for IR.

## Supplementary information

**Additional file 1: Figure S1.** Weight changes. Weekly weight changes in each group of rats from 0 week to 12 weeks.

**Additional file 2: Figure S2.** The Oral glucose tolerance test. Fig. A shows the OGTT curve, Fig. B shows the area under curve of the oral glucose tolerance test (OGTT) of each experimental group. Compared with the model group, # *P* < 0.05, ## *P* < 0.01, and ### *P* < 0.001.

**Additional file 3: Figure S3.** the uncropped microscopy images of liver specimens.

**Additional file 4: Figure.S4.** the uncropped blot images of the protein expression of GPR43, p-AMPK and GAPDH in liver and muscle. In the same experiment, the efficacy of other drugs was also tested, which will not explaine in this paper, so there are redundant bands in some images. The red box indicates the final cropping range.

## Data Availability

The datasets used and/or analyzed for this study are available from the corresponding author by reasonable request.

## Abbreviations

AMPK: Adenosine 5′-monophosphate -activated protein kinase
CON: Control group
FBG: Fasting blood glucose
FGC: Fasting glucagon
GPR43: G protein-coupled receptor 43
HDL-C: High-density lipoprotein cholesterol
IR: Insulin resistance
LDL-C: Low density lipoprotein cholesterol
MET: Positive control group
MOD: Model group
MS: Metabolic syndrome.
NAFLD: Non-alcoholic fatty liver disease
PPM: Processed *Polygonum multiflorum*
PPM-H: Water extraction group of PPM (high dose groups)
PPM-L: Water extraction group of PPM (low high dose groups)
Sensn2: Stress-inducible protein Sestrin2
SD: Sprague Dawley
TC: Total cholesterol
TG: Triglyceride
