# Supplementary figures and images for: Water extract from processed Polygonum multiflorum modulate gut microbiota and glucose metabolism on insulin resistant rats
Source: BMC Complement Med Ther. 2020 Apr 5;20:107. doi: 10.1186/s12906-020-02897-5 (PMC7132990; doi:10.1186/s12906-020-02897-5)

(A) normal control group


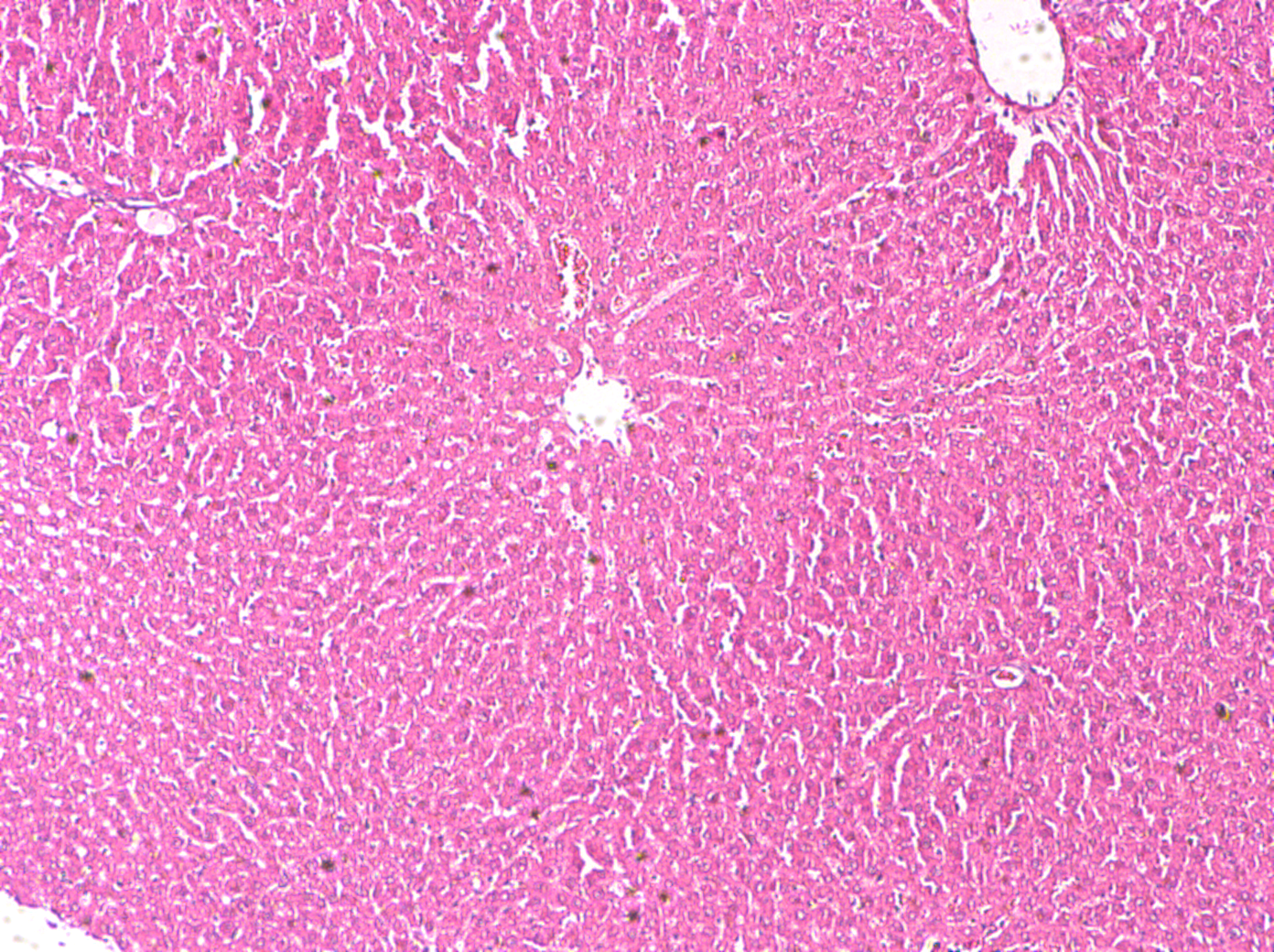
(B) model control group


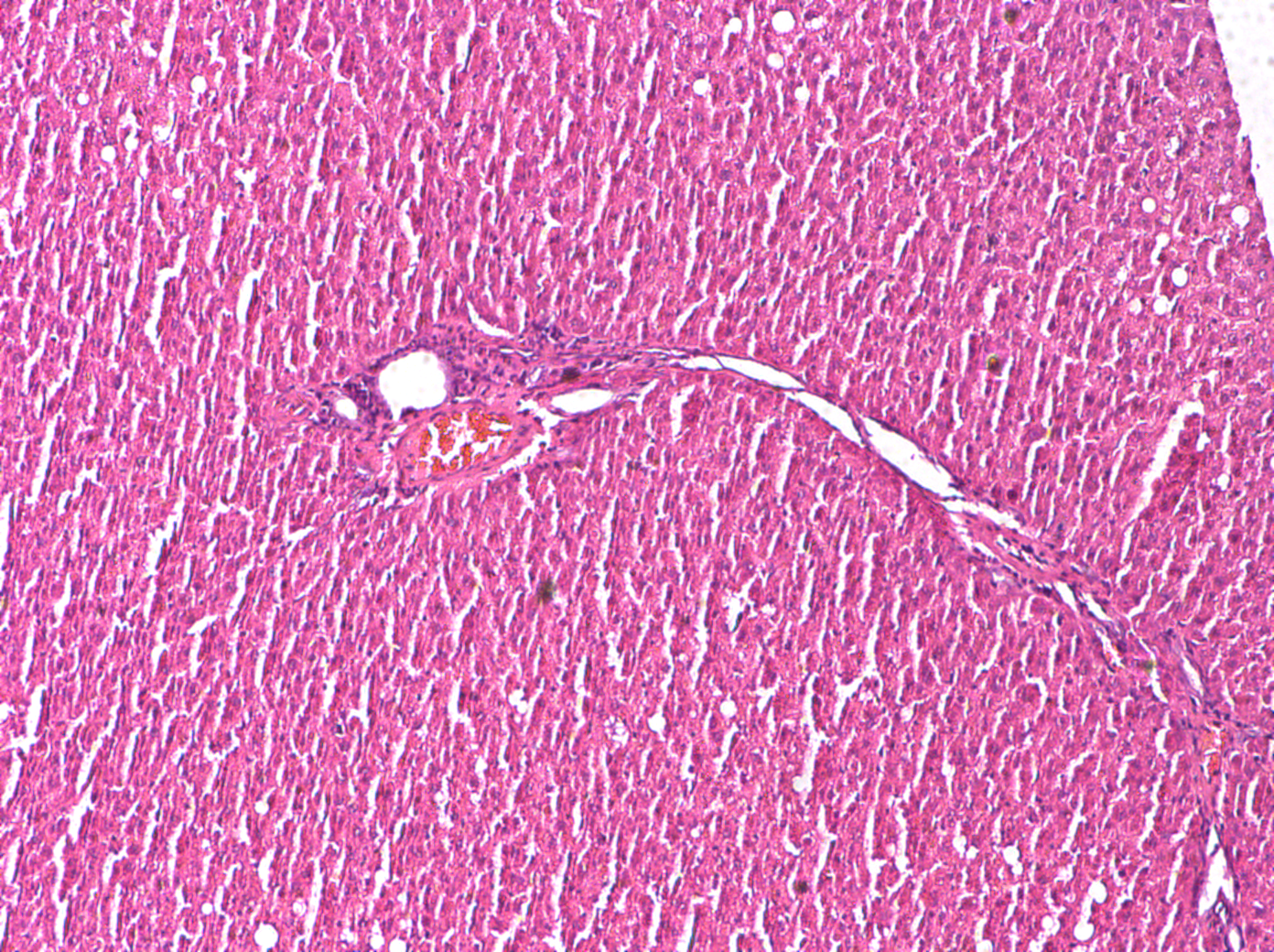


(C) positive control group


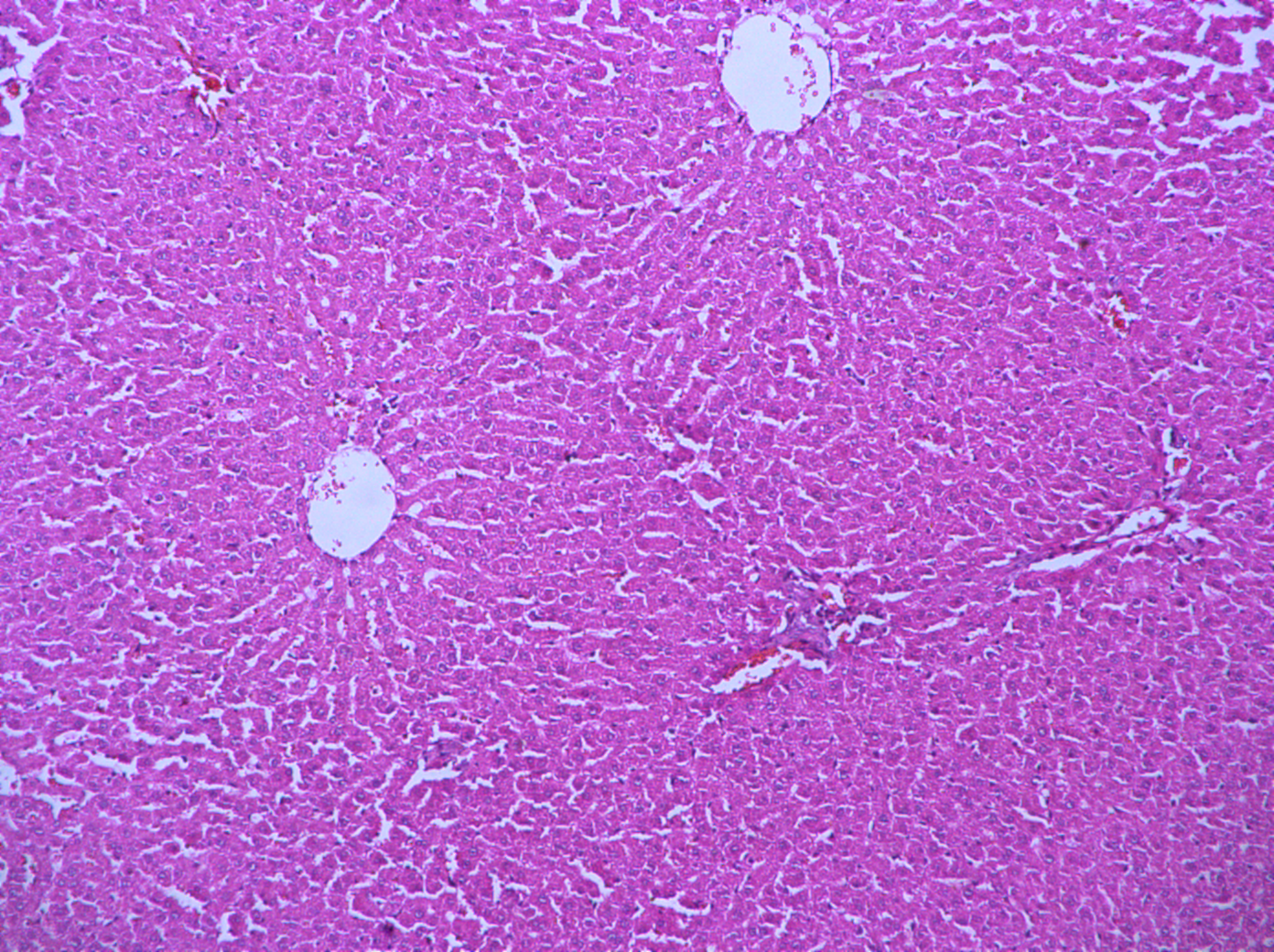


(D) PPM-L group


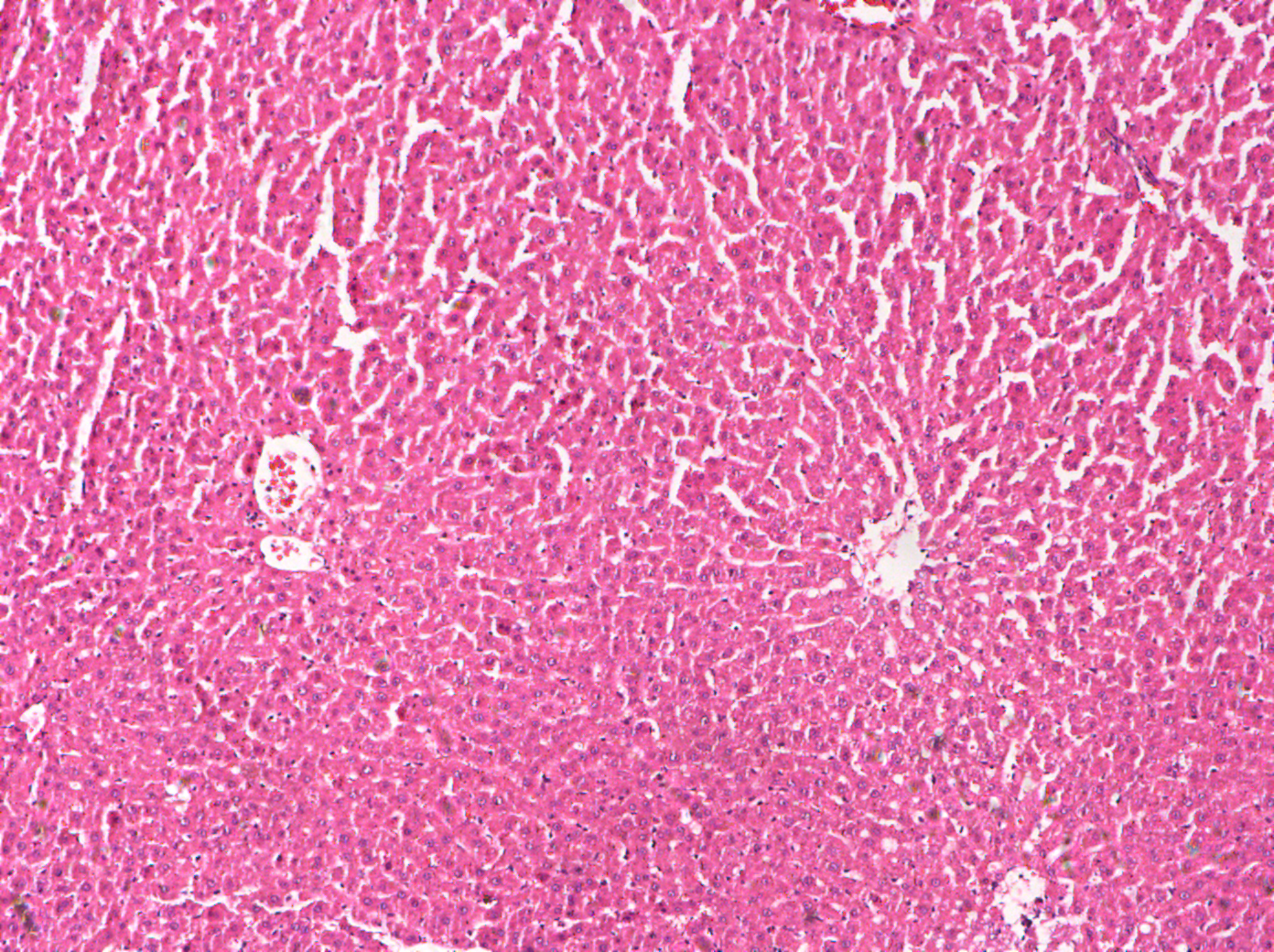


(E) PPM-L group


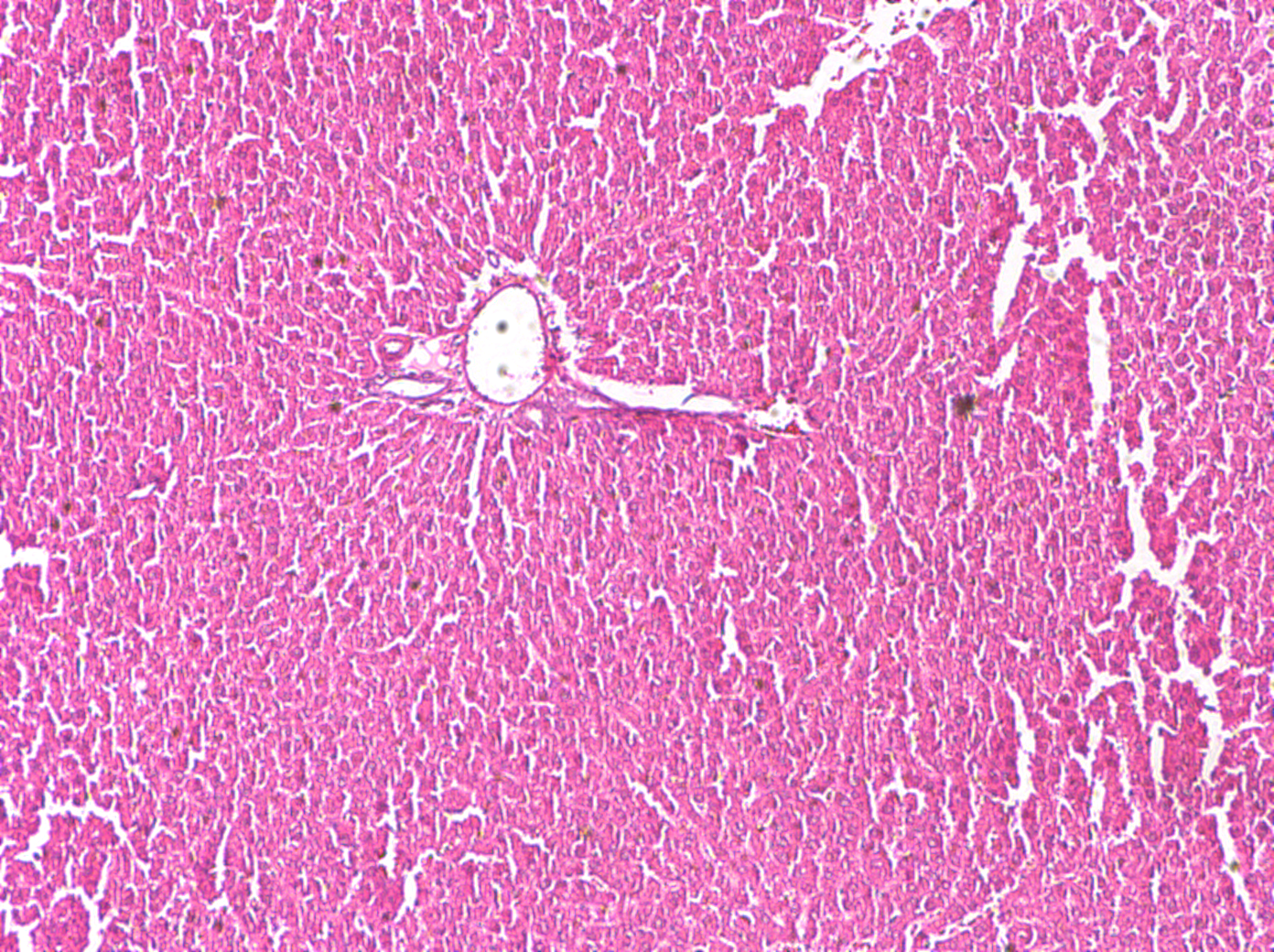

Supplement: Supplementary file 3 — Additional file 3: Figure S3. the uncropped microscopy images of liver specimens. [file 12906_2020_2897_MOESM3_ESM.doc]
