# Supplementary material for: Water extract from processed Polygonum multiflorum modulate gut microbiota and glucose metabolism on insulin resistant rats
Source: BMC Complement Med Ther. 2020 Apr 5;20:107. doi: 10.1186/s12906-020-02897-5 (PMC7132990; doi:10.1186/s12906-020-02897-5)

1. the protein expression of GPR43 in liver


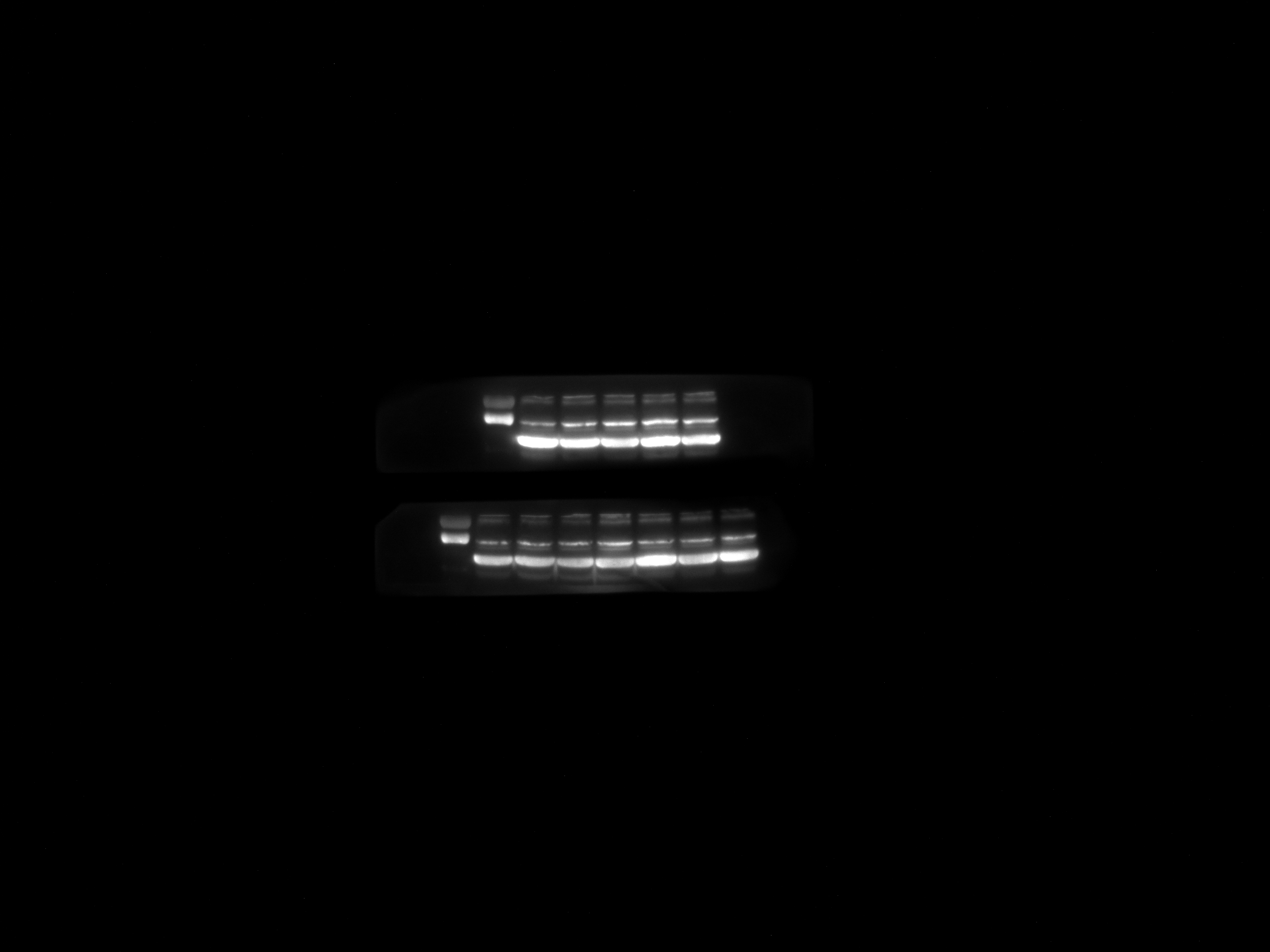


1. the protein expression of GPR43 in muscle


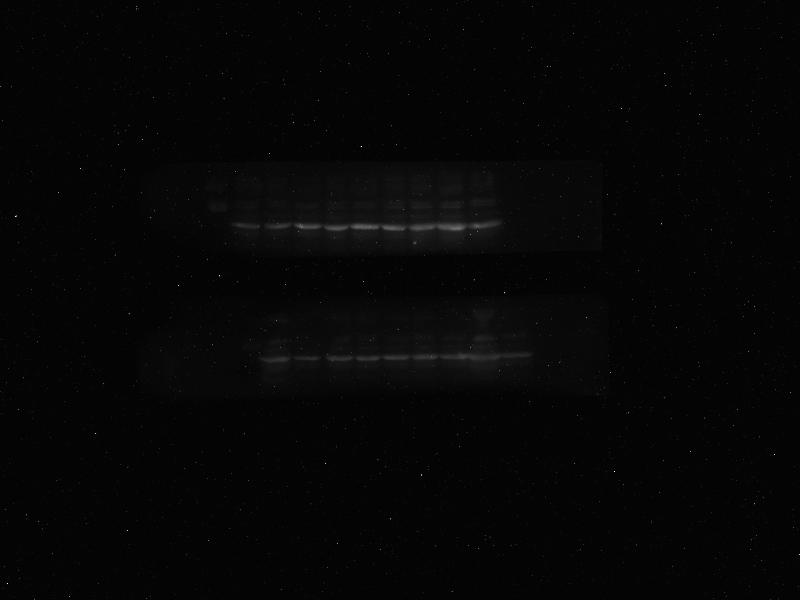


1. the protein expression of p-AMPK in liver


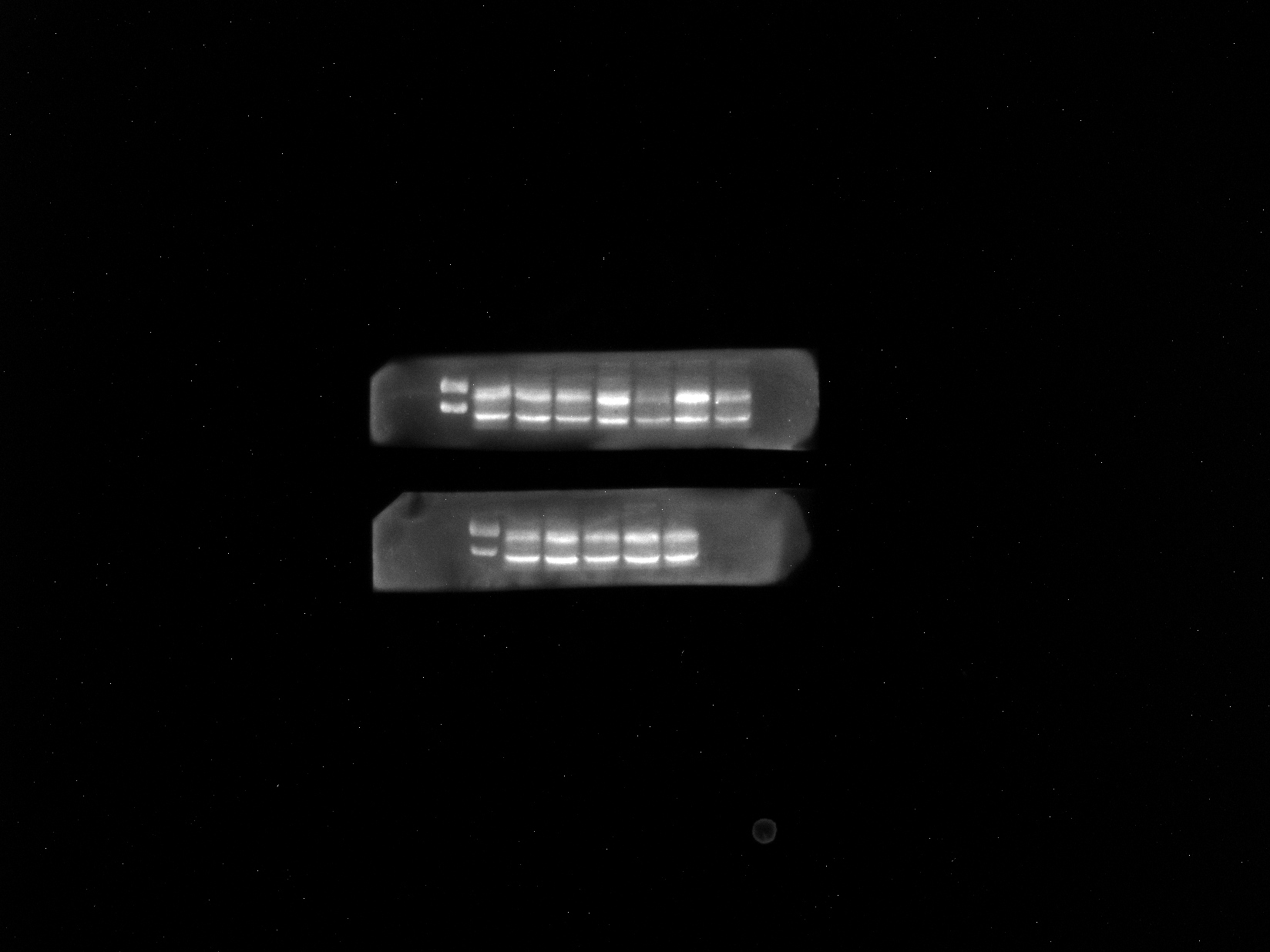


1. the protein expression of p-AMPK in muscle


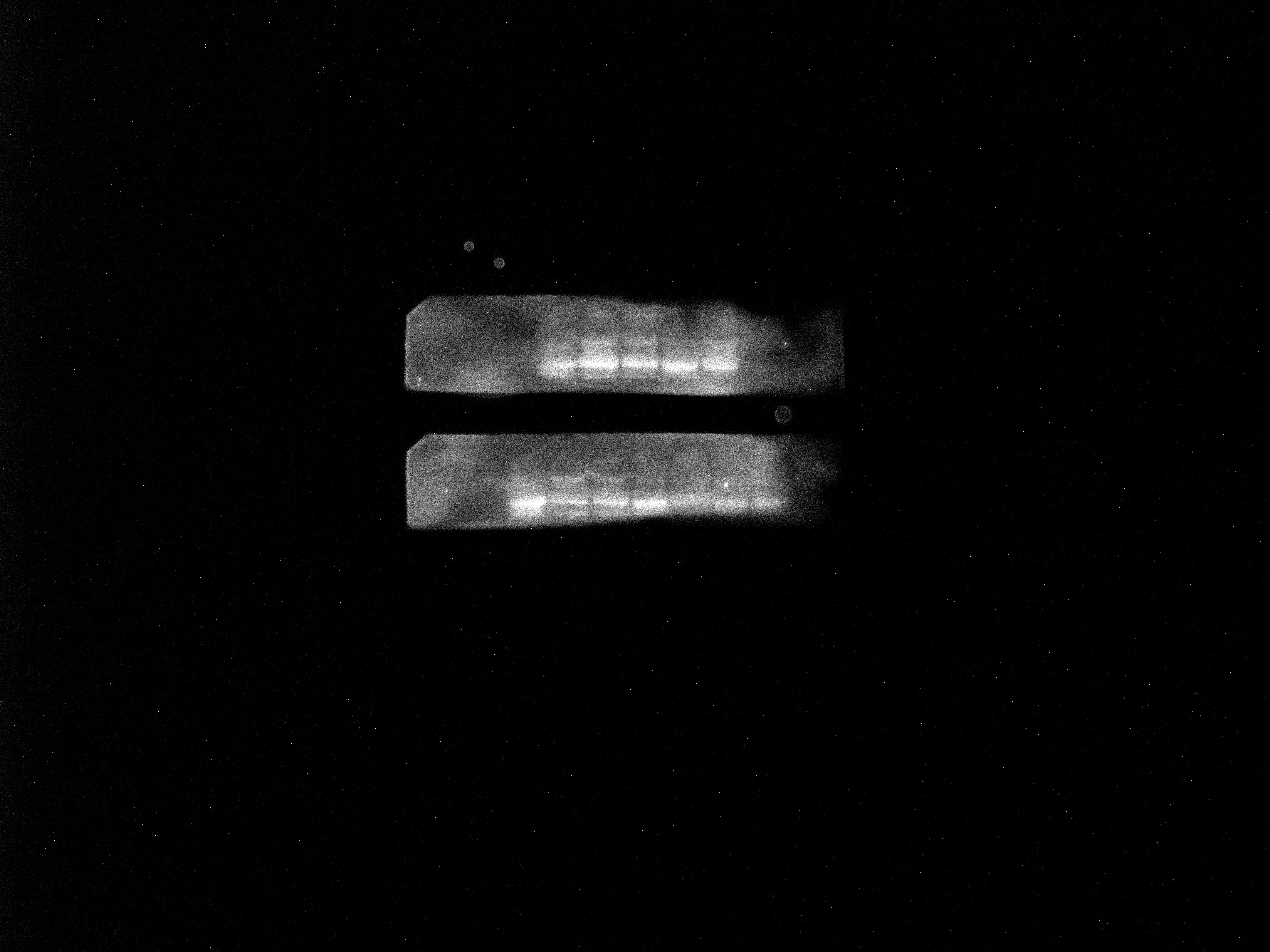


1. the protein expression of GAPDH in liver


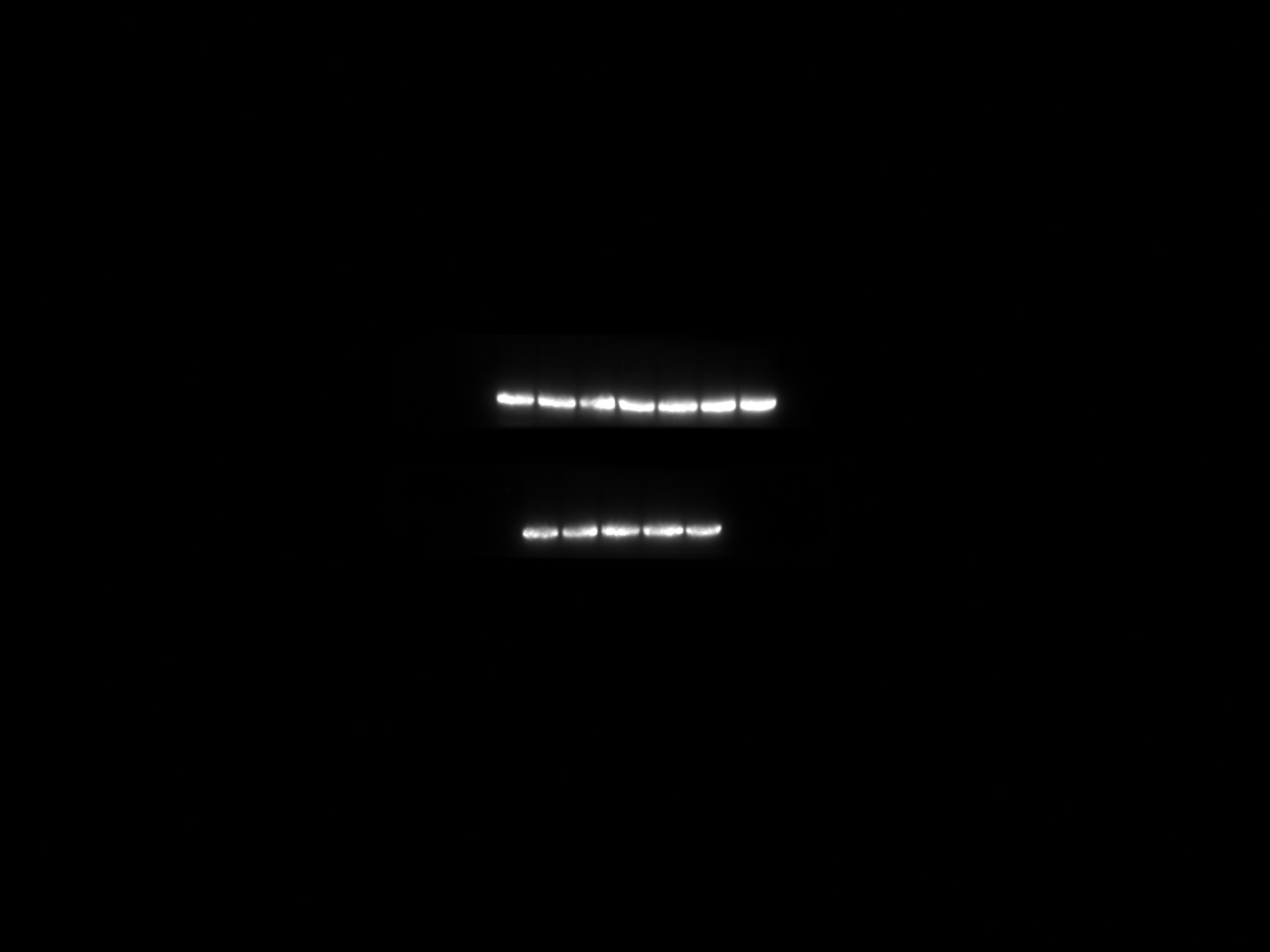


(F) the protein expression of GAPDH in muscle


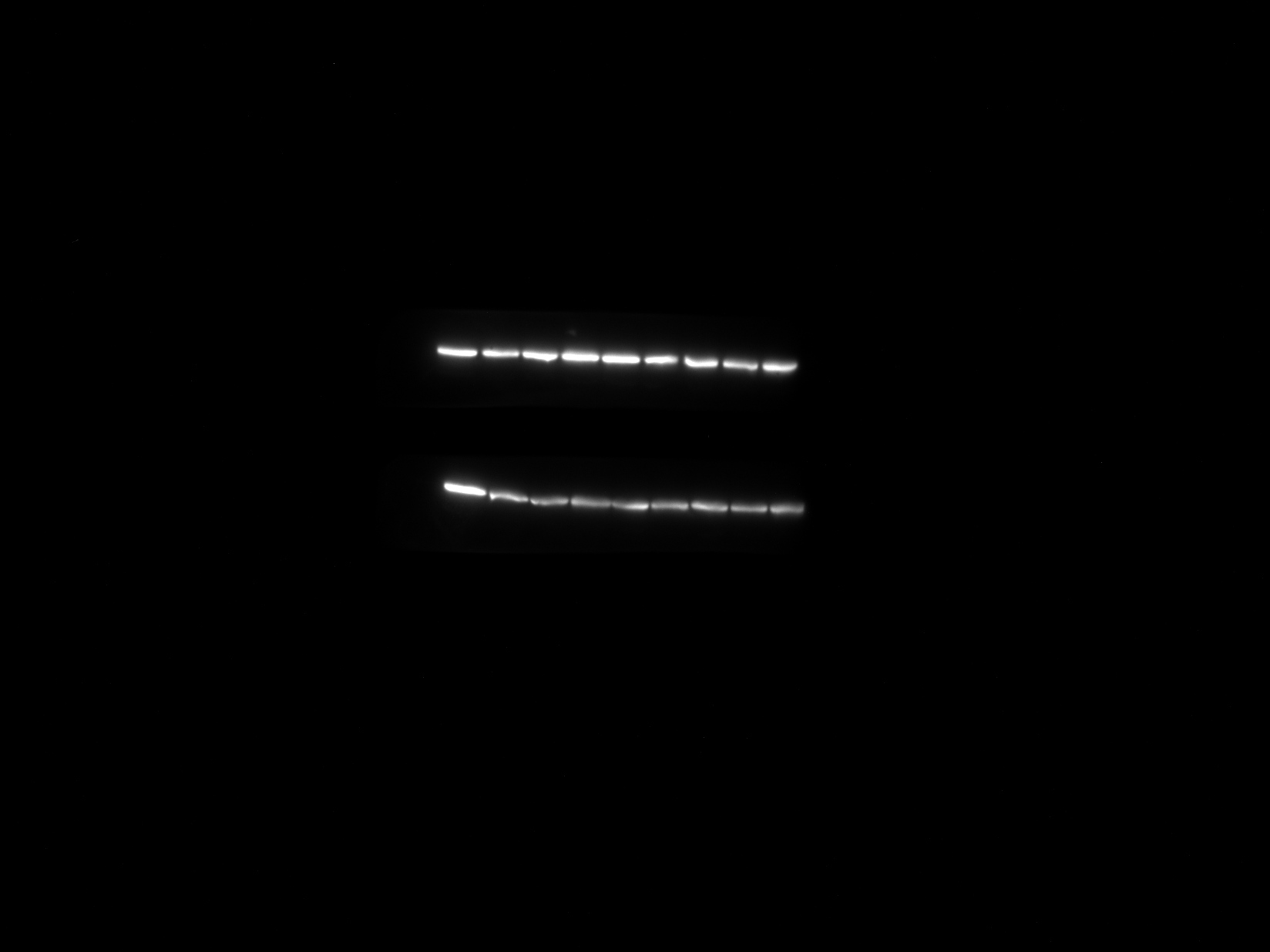

Supplement: Supplementary file 4 — Additional file 4: Figure.S4. the uncropped blot images of the protein expression of GPR43, p-AMPK and GAPDH in liver and muscle. In the same experiment, the efficacy of other drugs was also tested, which will not explaine in this paper, so there are redundant bands in some images. The red box indicates the final cropping range. [file 12906_2020_2897_MOESM4_ESM.doc]
