# Supplementary material for: Water extract from processed Polygonum multiflorum modulate gut microbiota and glucose metabolism on insulin resistant rats
Source: BMC Complement Med Ther. 2020 Apr 5;20:107. doi: 10.1186/s12906-020-02897-5 (PMC7132990; doi:10.1186/s12906-020-02897-5)
Supplement: Supplementary file 3 [file 12906_2020_2897_MOESM1_ESM.doc]

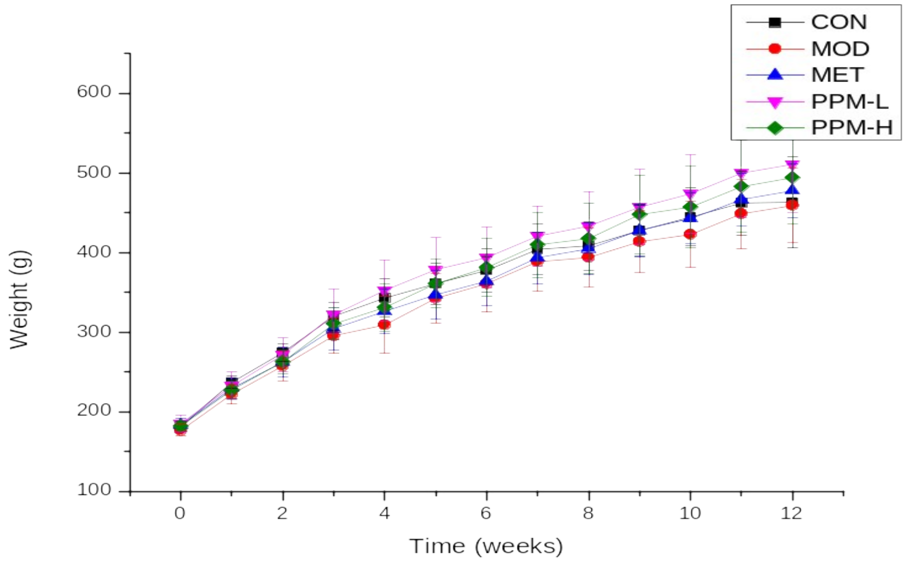

CON
MOD
MET
PPM-L
PPM-H
600
500
400
300
200
100
Weight (g)
0
2
4
6
8
10
12
Time (weeks)
